# *Helicobacter pylori* CagA Suppresses Apoptosis through Activation of AKT in a Nontransformed Epithelial Cell Model of Glandular Acini Formation

**DOI:** 10.1155/2015/761501

**Published:** 2015-10-18

**Authors:** Gabriela Vallejo-Flores, Javier Torres, Claudia Sandoval-Montes, Haruki Arévalo-Romero, Isaura Meza, Margarita Camorlinga-Ponce, Julián Torres-Morales, Adriana Karina Chávez-Rueda, María Victoria Legorreta-Haquet, Ezequiel M. Fuentes-Pananá

**Affiliations:** ^1^Departamento de Inmunología, Escuela Nacional de Ciencias Biológicas, Instituto Politécnico Nacional, Prolongación de Carpio, s/n, Colegio Santo Tomas, 11340 México, DF, Mexico; ^2^Unidad de Investigación Médica en Enfermedades Infecciosas y Parasitarias (UIMEIP), Hospital de Pediatría, CMN Siglo-XXI, Instituto Mexicano del Seguro Social (IMSS), Avenida Cuauhtémoc No. 330, Colegio Doctores, 06720 México, DF, Mexico; ^3^Unidad de Investigación en Virología y Cáncer, Hospital Infantil de México Federico Gómez, Dr. Márquez 162, Colegio Doctores, 06720 México, DF, Mexico; ^4^Departamento de Biomedicina Molecular, Centro de Investigación y Estudios Avanzados del Instituto Politécnico Nacional, Avenida Instituto Politécnico Nacional No. 2508, Colegio San Pedro Zacatenco, 07360 México, DF, Mexico; ^5^Facultad de Química, Universidad Nacional Autónoma de México, Avenida Universidad No. 3000, Colegio Universidad Nacional Autónoma, 04510 México, DF, Mexico; ^6^Unidad de Investigación Médica en Inmunología, Hospital de Pediatría, CMN Siglo-XXI, Instituto Mexicano del Seguro Social (IMSS), Avenida Cuauhtémoc No. 330, Colegio Doctores, 06720 México, DF, Mexico

## Abstract

*H. pylori* infection is the most important environmental risk to develop gastric cancer, mainly through its virulence factor CagA. *In vitro* models of CagA function have demonstrated a phosphoprotein activity targeting multiple cellular signaling pathways, while cagA transgenic mice develop carcinomas of the gastrointestinal tract, supporting oncogenic functions. However, it is still not completely clear how CagA alters cellular processes associated with carcinogenic events. In this study, we evaluated the capacity of *H. pylori* CagA positive and negative strains to alter nontransformed MCF-10A glandular acini formation. We found that CagA positive strains inhibited lumen formation arguing for an evasion of apoptosis activity of central acini cells. In agreement, CagA positive strains induced a cell survival activity that correlated with phosphorylation of AKT and of proapoptotic proteins BIM and BAD. Anoikis is a specific type of apoptosis characterized by AKT and BIM activation and it is the mechanism responsible for lumen formation of MCF-10A acini *in vitro* and mammary glands *in vivo*. Anoikis resistance is also a common mechanism of invading tumor cells. Our data support that CagA positive strains signaling function targets the AKT and BIM signaling pathway and this could contribute to its oncogenic activity through anoikis evasion.

## 1. Introduction

*Helicobacter pylori *(*H. pylori*) colonizes the human gastric epithelium and is considered the most important cause of chronic active gastritis, peptic ulcer, and gastric cancer [1]. The pathogenesis of* H. pylori* is importantly associated with the presence of the cag pathogenicity island (cagPAI) and the* cagPAI* effector protein, the cytotoxin-associated gene A (CagA) [2]. The* cagPAI* is a segment of DNA of about 40 kb that encodes a type IV secretion system (T4SS), which is necessary for CagA translocation into target epithelial cells. Once inside the cell, CagA is phosphorylated in tyrosine residues of the EPIYA motif by host cytoplasmic Src and c-Abl kinases, and phosphorylated and nonphosphorylated CagA interact with multiple signaling proteins [3–8].

*H. pylori* activation of the phosphoinositide 3-kinase (PI3K) and protein kinase B (PKB/AKT) signaling pathway has been previously documented in transformed gastric epithelial cells (AGS cells), although the mechanism by which this happens is not fully understood. On one hand, some studies support CagA phosphorylation dependent and independent roles [9–11]. On the other hand, a role for proinflammatory outer membrane (OipA) and vacuolating cytotoxin A (VacA) proteins has been proposed [12, 13], ruling out a role for the* cagPAI* [14]. Also, multiple targets downstream of PI3K/AKT have been documented, including mammalian target for rapamycin (mTOR), forkhead box O (FoxO)-1 and -3a ERK mitogen activated kinase, and proapoptotic protein BAD [15–19]. Concordantly, the consequence of* H. pylori* activation of PI3K/AKT is also unclear, with different studies supporting deregulation of apoptosis, proliferation, or cell migration.

The use of transformed cells has been essential to understand* H. pylori* pathogenesis, but it may also contribute to the conflicting data as many signaling pathways and cellular processes associated with cell transformation are already deregulated. CagA-induced proliferation and altered cell polarity have also been shown in nontransformed Madin-darby canine kidney epithelial cells (MDCK cells), but CagA's signaling has been partially described [20, 21]. It was reported that CagA disrupts epithelial apical-basolateral polarity in MDCK cells by interacting with PAR1/MARK kinase, which prevents atypical protein kinase C- (aPKC-) mediated PAR1 phosphorylation [22]. More definitive evidence of the CagA oncogenic role comes from transgenic mice, in which CagA expression induced epithelial hyperplasia, polyp formation, and adenocarcinomas of the gastrointestinal tract [23, 24]. Also, CagA transgenic expression in zebrafish induced epithelial cell proliferation and upregulation of cyclin D1, axin2, and the c-myc ortholog myca [25].

To better understand CagA interactions with cancer-associated signaling pathways and cellular processes, we studied CagA activity in a model of nontransformed epithelial cells. The epithelial cell line MCF-10A forms three-dimensional (3D) acini-like spheroids with a hollow lumen and an apicobasal orientation when cultured in a simile of the extracellular matrix (ECM). These characteristics allow testing mechanisms of cell proliferation, cell survival and the cytoskeletal structure that yields the polarized spheroid architecture [26, 27]. Hence, this 3D cellular system has been previously used to test cellular and viral oncogenes and has proved useful to decipher mechanisms of transformation and their targeted cellular signaling pathways [28, 29]. We infected MCF-10A spheroids with CagA positive and negative* H. pylori* variants finding that CagA positive strains caused evasion of apoptosis that was associated with phosphorylation of AKT, BIM, and BAD, which suggests that CagA inhibits the anoikis form of apoptosis.

## 2. Material and Methods 

### 2.1. *Helicobacter pylori* Strains and Culture

Two CagA positive* H. pylori* strains were used in this study: strain 11637 with a Western-type CagA (EPIYA ABCCC) that was obtained from the American Type Culture Collection (ATCC, Manassas, VA, USA No. 43504); and strain NY02-149 with an East-Asian-type CagA (EPIYA ABD) that was kindly donated by Dr. Guillermo Perez-Perez from New York University. Two additional* H. pylori* CagA negative variants were used as controls: strain 365A3, which has a partial* cagPAI* lacking the effector protein CagA and strain 254 that contains a nonfunctional cagPAI [30]. The latter will be referred as the cagPAI negative strain. The two CagA positive strains and the CagA negative variant are* VacA* s1/m1, while the* cagPAI* negative strain is positive to s1 and negative to the m region (Data no show). All* H. pylori* strains were grown on blood agar (BD, Bioscience, San Jose, CA, USA No. 211037) for 48 h at 5% CO_2_ and 37°C.

### 2.2. PCR Assay

**Figure 1:**
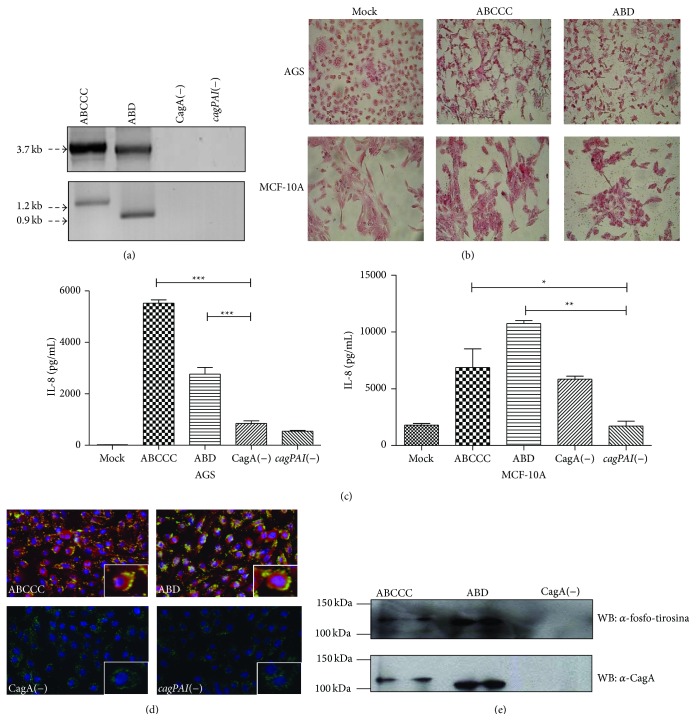
CagA is translocated into MCF-10A cells. (a) PCR amplification of the C-terminal variable region. (b) Morphology of AGS and MCF-10A cells infected with CagA positive strains. (c) IL-8 determination in supernatants of infected cells. (d) Immunofluorescence image showing* H. pylori* (green), CagA (red), and nuclei (Blue). Inbox is an optical zoom of an infected cell. (e) Whole lysates from infected MCF-10A cell were immunoprecipitated with anti-CagA antibodies and immunoblotted against total phosphotyrosine proteins (top panel). The blots were reprobed with anti-CagA and anti-*β*-actin antibodies. Statistical analysis by one-way ANOVA followed by the Tukey test (^*^ *p* ≤ 0.05, ^**^ *p* ≤ 0.01 and ^***^ *p* ≤ 0.001).

DNA was obtained from* H. pylori* strains using a Qiagen DNA extraction kit (Qiagen, Hilde, Germany No. 51306) according to the manufacturer protocols. DNA samples were subjected to PCR with primers cagA-F1 5′-ATGACTAACGAAACTATTGATCAA-3′ and cagA-R7 5′-TTAAGATTTTTGGAAACCAC-3′ for full-length* cagA* amplification and cagA-F5 5′-CCCTAGTCGGTAATGGGTTATC-3′ and CagA-R7 for EPIYA region amplification (Figure 1(a)). PCRs for VacA were also performed with primers VA1F 5′-ATGGAAATACAACAAACACAC-3′ and VA1R 5′-CTGCTTGAATGCGCCAAAC-3′ for amplification of the S region and VAGF 5′-CAATCTGTCCAATCAAGCGAG-3′ and VAGR 5′-GCGTCAAAATAATTCCCAAGG-3 for the M region.

### 2.3. Cells Lines and Infection

MCF-10A (CRL-10317) and AGS (CRL-1739) cells were obtained from the American Type culture collection (ATCC, Manassas, VA, USA). MCF-10A cells were grown in DMEM/F12 medium supplemented with 5% horse serum (Invitrogen, Carlsbad, CA, USA- No. 16050-114), 20 ng/mL of epidermal grown factor (PeproTech, Rocky Hill, NJ, USA. No AF-100-15), 5 *μ*g/mL hydrocortisone (No H-0808), 100 ng/mL cholera toxin (C-8052), and 10 *μ*g/mL insulin (I-1882) (all from Sigma Aldrich Co., St. Louis, MO, USA). AGS cells were grown in F12 medium supplemented with 10% fetal bovine serum (Invitrogen, Carlsbad, CA, USA, No. 16000044). All cultures also contained 100 units/mL of penicillin and 100 *μ*g/mL of streptomycin (Invitrogen, Carlsbad, CA, USA, No. 15240).

Infection was carried out with a MOI of 100 in MCF-10A and AGS cells at 80% subconfluency in nonsupplemented medium, unless otherwise specified. For proliferation assays MCF-10A cells were infected at 30% subconfluency in supplemented medium with 3% horse serum and the standard concentration of the other components. MCF-10A acini were formed in matrigel (BD Biosciences, San Jose, CA, USA, No. 354230) following the method reported by Debnath et al. [27]. 5 × 10^3^ MCF-10A cells were seeded in an 8-well plate (Lab-Tek Chamber Slide System, Nalge Nunc International, Rochester, NY, USA, No. 177402) on 40 *μ*L of a solidified layer of Matrigel. 400 *μ*L of culture medium was added per well and acini were allowed to form for 10–15 days, observing daily at the optical microscope and changing media every two days. Acini were grown in same medium as MCF-10A monolayers but with 2% of matrigel. For infection assays, MCF-10A cells were infected with a MOI of 100 in a monolayer for 2 h at 5% CO_2_ and 37°C before recovering the cells and seeding them in the layer of matrigel. Cells were grown for 10 to 15 days changing medium every other day. Media of days 2, 4, and 6 also contained bacteria at the same MOI. To inhibit CagA phosphorylation-dependent activity, the PP2 Src kinase inhibitor was added on day 0, 2, 4, and 6 of acini formation and pictures were taken on day 10.

### 2.4. Hummingbird Phenotype and IL-8 Analyses

AGS and MCF-10A cells were plated in six-well plates (Corning Incoporated Life Sciences, Tewksbury, MA, USA, No. 3516) at a concentration of 4 × 10^5^ cells per well and infected with* H. pylori* strains for 24 h; supernatants were collected and cells were fixed with 70% methanol and stained with safranin (Hycel, D.F, México No. 541) to analyze the hummingbird phenotype. Levels of IL-8 were determined by an enzyme-linked immunosorbent assay (ELISA) using a sandwich ELISA kit (BD, Bioscience, San Jose, CA, USA, No. 555244) according to the manufacturer's instructions. Supernatants were collected and stored at −80°C.

### 2.5. Western Blot and Immunoprecipitation Assays

MCF-10A cells were infected with* H. pylori* strains. After 6 h of infection, cultures were treated with 200 ng/mL gentamicin (Invitrogen, Carlsbad, CA, USA, No. 15750-060) for another hour to eliminate bacteria. Cells were washed 3x with PBS and lysed with RIPA buffer (No. 20-188) supplemented with a cocktail of protease inhibitors (No. 20-201) (both from Millipore, MA, USA) and 2 mM sodium orthovanadate (Sigma Aldrich Co., St. Louis, MO, USA, No. 56508) for 10 min on ice. Cell lysates were separated in 8 or 12% polyacrylamide gels (SDS-PAGE), transferred into a PVDF membrane (Millipore, Billerica, MA, USA, No. IPVH00010), and blocked overnight in 2% fat-free dry milk (for assays of nonphosphorylated proteins) or 5% bovine serum albumin (BSA) (for phosphorylated proteins) in TBS containing 0.1% Tween-20. Membranes were probed with polyclonal anti-CagA antibody (B-300; No. sc-25766) or an anti-tyrosine-phosphorylated protein antibody (PY99; No. sc-7020) (both from Santa Cruz Biotechnology) followed by the secondary antibody (Dako, Cambridge, UK, No. 201609). Blots were developed by chemiluminescence (Millipore, Billerica, MA, USA, No. WBKLS0100). Immunoprecipitations were performed using the anti-CagA antibody, followed by inmmunoblotting with the anti-CagA or anti-tyrosine-phosphorylated protein antibodies described above. Signaling pathways were analyzed after infection of either cell attached to flasks or growing in suspension, the latter to promote cell death and better visualize infection-induced protection. Cells were lysed at 30 min and 1, 2, 4, and 6 h after infection and inmmunoblots were performed as described above. The antibodies used for the assessment of signaling pathways were AKT (GTX121937), AKT-phosphorylated (GTX128414), BAD-phosphorylated (GTX50136), and BIM-phosphorylated (YE0911W) (all from GeneTex, Inc., San Antonio, Texas); a homemade *β*-actin antibody was kindly donated by Dr. Manuel Hernandez-Hernandez from CINVESTAV.

### 2.6. Immunofluorescence Assay

8 × 10^4^ MCF-10A cells were seeded on coverslips and infected with* H. pylori* strains for 6 h, fixed with paraformaldehyde (4% in PBS) for 10 min, and permeabilized with 0.2% Triton X-100 in PBS. Samples were incubated with blocking buffer (10% goat serum, 1% BSA, 0.2% triton X-100 and 0.05% Tween-20) for 1 h and then with a 1 : 500 dilution of a monoclonal anti-CagA antibody for 1 h (abcam, Cambridge, UK, No. ab37351) and with the secondary antibody anti-mouse-Cy3 for 30 min (Genetex No. GTX85338). Samples were reincubated with blocking buffer for 1 h and then with a 1 : 500 dilution of a polyclonal anti-*H. pylori* antibody (Dako, Cambridge, UK, No B0471) and with the secondary antibody anti-rabbit-FITC, each one for 30 min (Jackson ImmunoResearch, No. 711-095-152). Cells were observed using a fluorescence microscope Olympus BX51 and images were acquired with a digital camera (Camedia C4040, Olympus). For immunofluorescence analysis of 3D cultures the acini were fixed in 2% paraformaldehyde in PBS for 20 min at room temperature; cells were then permeabilized with PBS containing 0.5% Triton X-100 for 10 min at 4°C. After that, the acini were incubated with blocking buffer (1% BSA, 0.2% Triton X-100, 10% goat serum in PBS) for 1 h at room temperature and then overnight at 4°C with any of the following antibodies: a 1 : 300 dilution of anti-Ki67 antibody (Genetex, Cambridge, UK, No. GTX76072), a 1 : 500 dilution of a polyclonal anti-*H. pylori* antibody, and a 1 : 200 dilution of a monoclonal anti-CagA antibody (Abcam, Cambridge, UK, No. ab37351); and then the acini were incubated with the secondary antibody anti-rabbit-FITC or anti-mouse FITC (Sigma Aldrich Co., St. Louis, MO, USA., No. F0257), for 30 min. Finally, nuclei were stained with DAPI and acini were analyzed in a LSM5 Pascal confocal microscope (Zeiss). Serial confocal cross sections (*x-y* axis) were made in MCF-10A acini. See figures for schematic diagrams overlying each section, which illustrate the relative position of the optical section with respect to the* z-*axis.

### 2.7. Carboxyfluorescein Proliferation Assay

MCF-10A cells were labeled in a 12-well dish with 25 Mm Carboxyfluorescein (CFSE-Invitrogen, Carlsbad, CA, USA, No. 10023), according to the manufacturer's protocol. After labeling, cells were infected with the* H. pylori* strains and incubated 3 days at 37°C with 5% CO_2_. CFSE positive cells were quantified with BD CellQuest Pro software (BD Bioscience, Sparks, MD) using a FACS-Calibur flow cytometer (BD Bioscience, San Jose, CA).

### 2.8. Apoptosis Resistance Assay

MCF-10A cells were infected with* H. pylori *strains ABCCC (MOI = 100) and ABD (MOI = 25) in single-cell suspensions for 4 h with or without 8 *μ*M of the AKT inhibitor GSK690693 at 37°C. Cells were then stained with a KIT containing annexin V and propidium iodide (Biolegend, San Diego, CA, USA, No. 235678), according to the manufacturer's protocol. Annexin V and proprium iodide positive cells were quantified with the BD CellQuest Pro software using a FACS-Calibur flow cytometer.

### 2.9. Statistical Analysis

The statistical differences between two variables were determined with the Student's* t*-test; differences among three or more continuous variables were compared by one-way ANOVA, followed by the Tukey test. Statistical significance was established at* p* ≤ 0.05.

## 3. Results

### 3.1. *H. pylori* CagA Is Efficiently Translocated into Nontransformed MCF-10A Cells

The two CagA positive* H. pylori* strains that were used in our study were able to induce a hummingbird phenotype and IL-8 secretion in AGS cells, as previously described [31, 32], which are indirect markers of CagA translocation into these cells (Figures 1(b) and 1(c)). AGS and MCF-10A infections with analysis of hummingbird phenotype and IL-8 secretion were run in parallel through all experiments to monitor the viability of the* H. pylori* strains. To determine if MCF-10A cells were permissive to* H. pylori* infection, infected cells were analyzed by immunofluorescence and Western blot. The immunofluorescence analysis showed that* H. pylori*, regardless of CagA status, adhered to the cell membrane of MCF-10A, with CagA signal observed within the cell (Figure 1(d)). Detection of phosphorylated-CagA confirmed its cellular translocation and activation (Figure 1(e)). Of note, the CagA-ABD strain showed stronger intensity of the phosphorylated CagA band and stronger activity than the CagA-ABCCC strain. These results indicated that MCF-10A cells are permissive for infection/CagA translocation with* H. pylori* strains.

### 3.2. MCF-10A Cells Do Not Increase Proliferation in Response to* H. pylori *Infection

**Figure 2:**
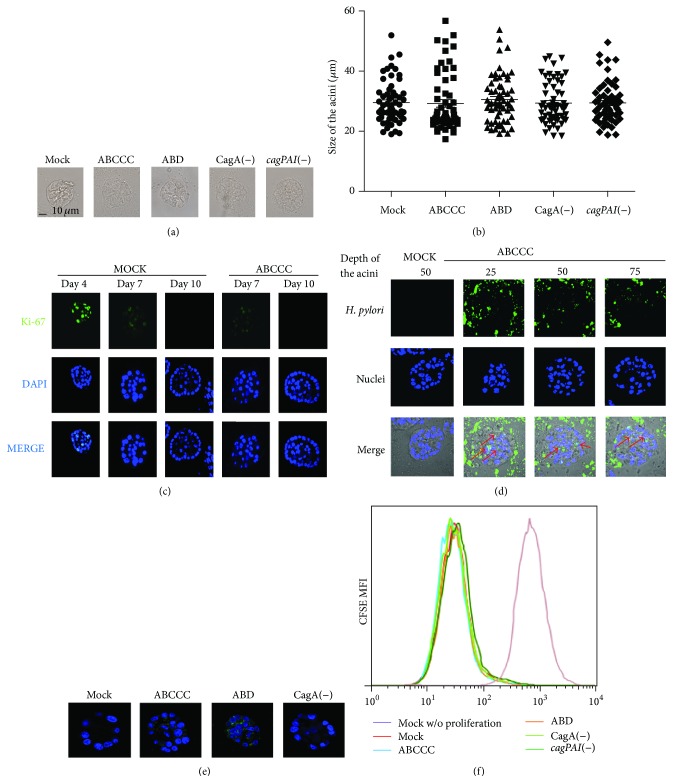
*H. pylori* does not induce proliferation of MCF-10A cell. MCF-10A cells were 3D cultured and infected with* H. pylori* strains. (a) Example of the average size of the acini. (b) The size of acini from three independent experiments was quantified at day 10, and no statistical differences were found. (c) 3D MCF-10A cells were stained with anti-Ki-67 antibody (green) and DAPI (blue) at different days of acini growth. (d) Confocal microscopy sections were made at 25, 50, and 75% deep of the acini. Red arrows indicate* H. pylori* (green) presence in close contact with the acini luminal cells (DAPI). (e) Confocal microscopy sections were made at 50% deep of the acini; image shows CagA (green) and nuclei (DAPI) in the acini central cells. (f) Monolayers of MCF-10A cells labeled with CFSE were infected with* H. pylori* strains for 3 days. No statistical differences were found in the rate of proliferation between the different infected cells.

In spite of multiple evidence supporting increased proliferation rates after* H. pylori* infection of transformed cells, infected MCF-10A acini showed normal size and maintained the spheroid structure (Figures 2(a) and 2(b)). Cells within the acini normally divide until day 7-8, after which further growth is arrested. To further test whether CagA translocation affected MCF-10A cell proliferation, infected acini were stained with an antibody against the proliferation marker Ki-67.  Figure 2(c) shows that both mock infected and CagA positive infected acini presented a dim Ki-67 signal at day seven and a negative signal by day ten, suggesting that* H. pylori* infection did not alter rates of acini growth. A day four staining is shown as a positive control for a bright Ki-67 signal. Nonetheless,* H. pylori* staining and confocal microscopy analysis of acini grown for 10 days show bacteria (Figure 2(d)) and CagA (Figure 2(e)) in close contact with external and central luminal cells, arguing in favor of sustained CagA translocation during acini growth. To confirm that* H. pylori* does not induce proliferation, CFSE-labeled MCF-10A cells were infected in monolayer for 3 days and CFSE fluorescence intensities were analyzed by flow cytometry (Figure 2(f)). No differences were observed in the rate of proliferation between cells infected with CagA positive and CagA negative variant strains.

### 3.3. MCF-10A Cells Evade Apoptosis after Infection with CagA Positive* H. pylori* Strains

**Figure 3:**
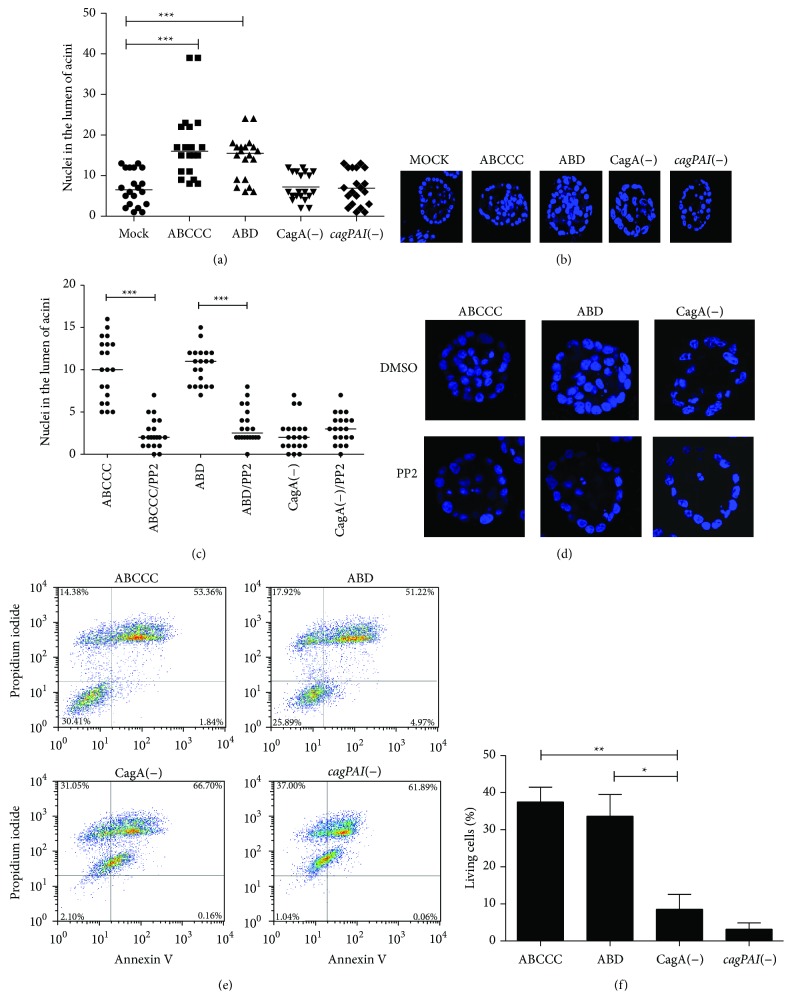
CagA induces evasion of apoptosis. (a) The nuclei (DAPI positive cells) in the lumen of acini were quantified on day 10. Each point represents one acini; three fields were counted in three independent experiments. (b) Representative acini showing the typical number of nuclei positive cells in lumens. (c) The same experiment was carried out in the presence of the Src kinase inhibitor PP2. (d) Representative acini showing the typical number of nuclei positive cells in lumens after PP2 treatment. (e) MCF-10A cells were infected in suspension with* H. pylori* strains and apoptosis was determined by anexin V and propidium iodide (PI) staining. (f) Average percentage of living cell (Anexin V & PI negative) from three independent experiments. Asterisks denote statistical differences (^*^ *p* ≤ 0.05, ^**^ *p* ≤ 0.01, and ^***^ *p* ≤ 0.001). (a) and (f) One-way ANOVA followed by the Tukey test, and (c) Student's* t*-test.

A consistent observation of MCF-10A acini infected with CagA positive* H. pylori* strains was the lack of a well-formed lumen. To explore the possibility that CagA induces survival of acini central cells, the number of nuclei was quantified at day 10 acini. Confocal microscopy transversal cuts 50% deep in the acini were used for nuclei count, finding significant differences between CagA positive* H. pylori* strains and mock infected acini (Figures 3(a) and 3(b)). Acini morphogenesis in the presence of the Src kinases inhibitor PP2 indicated that the survival of the acini central cells was dependent on phosphorylation of CagA (Figures 3(c) and 3(d)). MCF-10A cells were also infected in suspension and apoptosis was determined by Anexin V and propidium iodide (PI) staining and flow cytometry analysis. Figures 3(e) and 3(f) show that, after 6 h of suspension, cells infected with CagA negative variants were mostly dead, while about 40% of the ones infected with CagA positive strains remained anexin V and PI negative. Overall, these data suggest that CagA promotes survival of cells that have lost substrate or ECM interactions.

### 3.4. AKT and BIM Are Important Targets during CagA Positive* H. pylori* Mediated Evasion of Apoptosis

**Figure 4:**
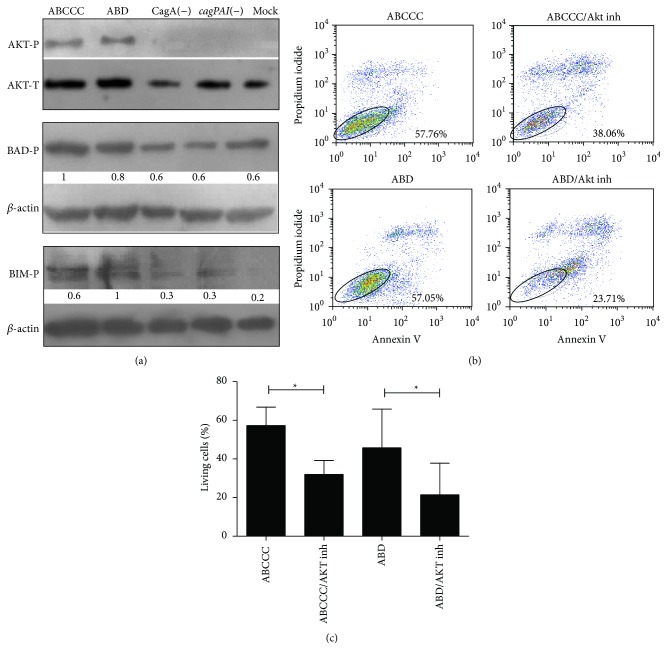
AKT induced evasion of apoptosis correlates with BIM and BAD phosphorylation. (a) MCF-10A cell lysates were immunoblotted against AKT, BIM, and BAD and their phosphorylated forms (AKT in Ser473, BAD in Ser136, and BIM in Ser87). A densitometric analysis of phosphorylated proteins was normalized to the corresponding *β*-actin levels. An arbitrary value of 1 was assigned to the highest activity. (b) MCF-10A cells were infected with* H. pylori* strains in cell suspension and an AKT inhibitor was added; apoptosis was determined by anexin V and propidium iodide (PI) staining. (c) Percentage of living cells (Anexin V and PI negative). Representative assay of three independent experiments is shown. Asterisks denote statistical differences by the Student's* t*-test, (^*^ *p* ≤ 0.05).

Anoikis is a form of apoptosis responsible for death of the acini luminal cells after loss of ECM interactions. Anoikis resistance in MCF-10A acini formation and mammary gland development has been associated with constitutive activity of the PI3K/AKT signaling pathway and phosphorylation-dependent inactivation of proapoptotic proteins mainly BIM but also BAD [33]. To evaluate whether CagA-induced activation of AKT was responsible for the anoikis resistance observed in MCF-10A acini luminal cells, we first tested AKT phosphorylation in response to* H. pylori* infection by Western blot analysis. MCF-10A cells were infected in single-cell suspensions with* H. pylori* strains for 4 h and cell lysates were blotted with the antiphosphorylated AKT.  Figure 4(a) shows the specific activation of AKT by CagA positive strains. Also, a preferentially increased phosphorylation of BIM over BAD was observed (Figure 4(a)). We then inhibited AKT activation reducing the frequency of live MCF-10A cells in suspension (Figures 4(b) and 4(c)). These results argue in favor of an* H. pylori* mechanism of anoikis evasion through CagA-induced activation of AKT and inhibition of BIM.

## 4. Discussion

The 3D culture of MCF-10A epithelial cells interacting with a simile of ECM results in formation of acini-like polarized structures that recapitulate many of the characteristics of the* in vivo* mammary gland architecture. Acini formation involves programmed grow arrest, anoikis of luminal cells, and preservation of the cytoskeleton-dependent morphology, biological and biological processes that are usually deregulated in cancer cells. Therefore, this system has been extensively used to study cellular and viral oncogenes, including some unrelated to mammary tissue but whose transforming mechanisms can be mirrored during acini formation [26, 28, 34–36].

Oncogene expression often results in MCF-10A acini with solid lumens, similar to the ones observed in this study after infection with CagA positive* H. pylori* strains, and this is considered an indication of resistance to anoikis [37, 38]. Anoikis is a form of apoptosis responsible for death of inner cell populations that have lost ECM interactions and grow factor stimulus, forming the hollow ducts in which milk is transported during mammary gland morphogenesis. MCF-10A* in vitro* acini formation has been critical to understand the* in vivo* mechanism of anoikis during mammary morphogenesis [26, 27]. Anoikis resistance is very important during cancer initiation and progression since it is most likely responsible for the survival capacity of tumor cells filling the luminal glandular space in early carcinomas and of detached invasive and metastatic tumor cell [39], including gastric cancer cells [40]. In agreement, bonafide oncoproteins such as ERBB2 and CFS1R induce resistance to anoikis facilitating migration and metastasis of malignant cells [41–43].

In this paper we have shown that CagA positive* H. pylori* strains activate AKT resulting in evasion of apoptosis, a finding that correlates with previous reports in AGS transformed gastric tumor cells [9–11]. AKT is an important regulator of cell survival. AKT is activated by PI3K leading to inactivation of various proapoptotic proteins, including BIM and BAD [19]. AKT phosphorylates BAD in Ser136 and BIM in Ser87 in response to cell-integrin interactions. Phosphorylated BAD and BIM are then sequestered by the 14-3-3 complex counteracting its proapoptotic activity [44, 45]. Loss of the AKT activity results in augmented apoptosis [38, 46], and activating mutations in PI3K and AKT are often found in gastric tumors [47]. Furthermore, lumen formation by mammary glands and MCF-10A cells tightly depends on BIM activity [33, 48].

Several studies also support an increased rate of cell apoptosis after* H. pylori* infection mediated by different bacterial factors, mainly VacA [49–51], gamma-glutamyl transpeptidase [52], the cagPAI [53], and CagA itself [54]. Cancer precursor gastric lesions, such as atrophic gastritis and metaplasia, are already characterized by loss of the glandular structure. These lesions may result from infection-triggered unbalanced cell apoptosis intimately linked to increased cellular regeneration to repair the gastric mucosa. CagA may help to counteract those apoptosis-inducing mechanisms triggered by bacterial factors or cell death resulting from chronic inflammation [55, 56].

Our results showed that* H. pylori* infection does not increase proliferation of MCF-10A cells during acini or monolayer growth, which contrasts with previous studies showing that phosphorylated CagA interacts with GRB2 or SHP2 activating the Ras/MAPK pathway, leading to cell scattering and cell proliferation [4, 57]. Other studies support that* H. pylori* promote proliferation through upregulating miR-222 [58]. In addition, zebrafish expressing transgenic CagA exhibited epithelial cell proliferation with significant upregulation of gene markers of proliferation* CCND1* and the c-myc ortholog myca [25]. Similarly, CagA transgenic mice showed gastric epithelial hyperplasia [23]. We have no clear explanation for these differences, but we hypothesize that this may be due to the different genetic background of the cell lines used in those studies and our study. While in animal models multiple processes converge during carcinogenesis, such as inflammation and persistent tissue damage due to chronic CagA expression.

Studies in nontransformed MDCK cells found that* H. pylori* induced loss of cell polarity and mobilization of zonula occludens-1 (ZO-1) protein with loss of integrity of the tight junctions [20, 21]. In contrast, we did not observe gross cytoskeletal changes in infected MCF-10A acini, which maintained the spheroid structure. Analysis of proteins participating in monolayer integrity and cell-to-cell contacts is needed together with nutrient deprivation studies to better understand the capacity of* H. pylori* and CagA to disturb these processes in MCF-10A acini.

The importance to test* H. pylori* pathogenicity in cellular models of organogenesis has been highlighted by the appearance of three recent papers in which mouse and human primary gastric organoids are tested for* H. pylori* infection and CagA signaling activity [59–61]. Two of those studies provide evidence of a CagA-induced epithelial to mesenchymal transition (EMT). Interestingly, EMT is importantly regulated by GSK3*β* inhibition of *β*-catenin and GSK3*β* is negatively regulated by AKT. These studies and ours place AKT as an important target of CagA induced carcinogenesis.

## 5. Conclusion

We found that* H. pylori *CagA positive strains induce anoikis resistance in MCF-10A acini through the AKT signaling pathway, via phosphorylation and inactivation of proapoptotic proteins BIM and BAD. This CagA-dependent mechanism of anoikis resistance may contribute to the* H. pylori*/CagA carcinogenic potential. Our results also support the use of nontransformed cells and* in vitro* organogenesis in order to better understand the oncogenic mechanisms involved in* H. pylori* associated cancer development.

## References

[1] Parsonnet J., Friedman G. D., Vandersteen D. P. (1991). *Helicobacter pylori* infection and the risk of gastric carcinoma. *The New England Journal of Medicine*.

[2] Parsonnet J., Friedman G. D., Orentreich N., Vogelman H. (1997). Risk for gastric cancer in people with CagA positive or CagA negative *Helicobacter pylori* infection. *Gut*.

[3] Higashi H., Tsutsumi R., Muto S. (2002). SHP-2 tyrosine phosphatase as an intracellular target of *Helicobacter pylori* CagA protein. *Science*.

[4] Mimuro H., Suzuki T., Tanaka J., Asahi M., Haas R., Sasakawa C. (2002). Grb2 is a key mediator of *Helicobacter pylori* CagA protein activities. *Molecular Cell*.

[5] Mueller D., Tegtmeyer N., Brandt S. (2012). c-Src and c-Abl kinases control hierarchic phosphorylation and function of the CagA effector protein in Western and East Asian *Helicobacter pylori* strains. *The Journal of Clinical Investigation*.

[6] Suzuki M., Mimuro H., Suzuki T., Park M., Yamamoto T., Sasakawa C. (2005). Interaction of CagA with Crk plays an important role in Helicobacter pylori-induced loss of gastric epithelial cell adhesion. *Journal of Experimental Medicine*.

[7] Tsutsumi R., Higashi H., Higuchi M., Okada M., Hatakeyama M. (2003). Attenuation of *Helicobacter pylori* CagA·SHP-2 signaling by interaction between CagA and C-terminal Src kinase. *The Journal of Biological Chemistry*.

[8] Selbach M., Paul F. E., Brandt S. (2009). Host cell interactome of tyrosine-phosphorylated bacterial proteins. *Cell Host and Microbe*.

[9] Nagy T. A., Frey M. R., Yan F., Israel D. A., Polk D. B., Peek R. M. (2009). *Helicobacter pylori* regulates cellular migration and apoptosis by activation of phosphatidylinositol 3-kinase signaling. *Journal of Infectious Diseases*.

[10] Tabassam F. H., Graham D. Y., Yamaoka Y. (2009). Helicobacter pylori activate epidermal growth factor receptor- and phosphatidylinositol 3-OH kinase-dependent Akt and glycogen synthase kinase 3*β* phosphorylation. *Cellular Microbiology*.

[11] Suzuki M., Mimuro H., Kiga K. (2009). *Helicobacter pylori* CagA phosphorylation-independent function in epithelial proliferation and inflammation. *Cell Host and Microbe*.

[12] Zhang J., Qian J., Zhang X., Zou Q. (2014). Outer membrane inflammatory protein A, a new virulence factor involved in the pathogenesis of *Helicobacter pylori*. *Molecular Biology Reports*.

[13] Nakayama M., Hisatsune J., Yamasaki E. (2009). Helicobacter pylori VacA-induced inhibition of GSK3 through the PI3K/Akt signaling pathway. *Journal of Biological Chemistry*.

[14] Sokolova O., Vieth M., Gnad T., Bozko P. M., Naumanna M. (2014). *Helicobacter pylori* promotes eukaryotic protein translation by activating phosphatidylinositol 3 kinase/mTOR. *The International Journal of Biochemistry & Cell Biology*.

[15] Tabassam F. H., Graham D. Y., Yamaoka Y. (2012). *Helicobacter pylori*-associated regulation of forkhead transcription factors FoxO1/3a in human gastric cells. *Helicobacter*.

[16] Zhu Y., Wang C., Huang J. (2007). The *Helicobacter pylori* virulence factor CagA promotes Erk1/2-mediated Bad phosphorylation in lymphocytes: a mechanism of CagA-inhibited lymphocyte apoptosis. *Cellular Microbiology*.

[17] Qi X. J., Wildey G. M., Howe P. H. (2006). Evidence that Ser87 of BimEL is phosphorylated by Akt and regulates BimEL apoptotic function. *The Journal of Biological Chemistry*.

[18] Zha J., Harada H., Yang E., Jockel J., Korsmeyer S. J. (1996). Serine phosphorylation of death agonist BAD in response to survival factor results in binding to 14-3-3 not BCL-X_L_. *Cell*.

[19] Zhang X., Tang N., Hadden T. J., Rishi A. K. (2011). *Akt, FoxO* and regulation of apoptosis. *Biochimica et Biophysica Acta—Molecular Cell Research*.

[20] Bagnoli F., Buti L., Tompkins L., Covacci A., Amieva M. R. (2005). *Helicobacter pylori* CagA induces a transition from polarized to invasive phenotypes in MDCK cells. *Proceedings of the National Academy of Sciences of the United States of America*.

[21] Tan S., Tompkins L. S., Amieva M. R. (2009). *Helicobacter pylori* usurps cell polarity to turn the cell surface into a replicative niche. *PLoS Pathogens*.

[22] Saadat I., Higashi H., Obuse C. (2007). Helicobacter pylori CagA targets PAR1/MARK kinase to disrupt epithelial cell polarity. *Nature*.

[23] Ohnishi N., Yuasa H., Tanaka S. (2008). Transgenic expression of *Helicobacter pylori* CagA induces gastrointestinal and hematopoietic neoplasms in mouse. *Proceedings of the National Academy of Sciences of the United States of America*.

[24] Miura M., Ohnishi N., Tanaka S., Yanagiya K., Hatakeyama M. (2009). Differential oncogenic potential of geographically distinct *Helicobacter pylori* CagA isoforms in mice. *International Journal of Cancer*.

[25] Neal J. T., Peterson T. S., Kent M. L., Guillemin K. (2013). *H. pylori* virulence factor CagA increases intestinal cell proliferation by Wnt pathway activation in a transgenic zebrafish model. *Disease Models and Mechanisms*.

[26] Debnath J., Brugge J. S. (2005). Modelling glandular epithelial cancers in three-dimensional cultures. *Nature Reviews Cancer*.

[27] Debnath J., Muthuswamy S. K., Brugge J. S. (2003). Morphogenesis and oncogenesis of MCF-10A mammary epithelial acini grown in three-dimensional basement membrane cultures. *Methods*.

[28] Debnath J., Mills K. R., Collins N. L., Reginato M. J., Muthuswamy S. K., Brugge J. S. (2002). The role of apoptosis in creating and maintaining luminal space within normal and oncogene-expressing mammary acini. *Cell*.

[29] Katz E., Lareef M. H., Rassa J. C. (2005). MMTV Env encodes an ITAM responsible for transformation of mammary epithelial cells in three-dimensional culture. *The Journal of Experimental Medicine*.

[30] Reyes-Leon A., Atherton J. C., Argent R. H., Puente J. L., Torres J. (2007). Heterogeneity in the activity of Mexican *Helicobacter pylori* strains in gastric epithelial cells and its association with diversity in the cagA gene. *Infection and Immunity*.

[31] Segal E. D., Cha J., Lo J., Falkow S., Tompkins L. S. (1999). Altered states: involvement of phosphorylated CagA in the induction of host cellular growth changes by *Helicobacter pylori*. *Proceedings of the National Academy of Sciences of the United States of America*.

[32] Sharma S. A., Tummuru M. K. R., Blaser M. J., Kerr L. D. (1998). Activation of IL-8 gene expression by *Helicobacter pylori* is regulated by transcription factor nuclear factor-kappa B in gastric epithelial cells. *The Journal of Immunology*.

[33] Mailleux A. A., Overholtzer M., Schmelzle T., Bouillet P., Strasser A., Brugge J. S. (2007). BIM regulates apoptosis during mammary ductal morphogenesis, and its absence reveals alternative cell death mechanisms. *Developmental Cell*.

[34] Hebner C., Weaver V. M., Debnath J. (2008). Modeling morphogenesis and oncogenesis in three-dimensional breast epithelial cultures. *Annual Review of Pathology: Mechanisms of Disease*.

[35] Grande S. M., Bannish G., Fuentes-Panana E. M., Katz E., Monroe J. G. (2007). Tonic B-cell and viral ITAM signaling: context is everything. *Immunological Reviews*.

[36] Singhal G., Leo E., Gadham Setty S. K., Pommier Y., Thimmapaya B. (2013). Adenovirus E1A oncogene induces rereplication of cellular DNA and alters DNA replication dynamics. *Journal of Virology*.

[37] Haenssen K. K., Caldwell S. A., Shahriari K. S. (2010). ErbB2 requires integrin *α*5 for anoikis resistance via Src regulation of receptor activity in human mammary epithelial cells. *Journal of Cell Science*.

[38] Isakoff S. J., Engelman J. A., Irie H. Y. (2005). Breast cancer-associated PIK3CA mutations are oncogenic in mammary epithelial cells. *Cancer Research*.

[39] Paoli P., Giannoni E., Chiarugi P. (2013). *Anoikis* molecular pathways and its role in cancer progression. *Biochimica et Biophysica Acta*.

[40] Uekita T., Tanaka M., Takigahira M. (2008). CUB-domain-containing protein 1 regulates peritoneal dissemination of gastric scirrhous carcinoma. *American Journal of Pathology*.

[41] Muthuswamy S. K., Li D., Lelievre S., Bissell M. J., Brugge J. S. (2001). ErbB2, but not ErbB1, reinitiates proliferation and induces luminal repopulation in epithelial acini. *Nature Cell Biology*.

[42] Kacinski B. M., Scata K. A., Carter D. (1991). FMS (CSF-1 receptor) and CSF-1 transcripts and protein are expressed by human breast carcinomas in vivo and in vitro. *Oncogene*.

[43] Schmelzle T., Hall M. N. (2000). TOR, a central controller of cell growth. *Cell*.

[44] Chiarugi P., Giannoni E. (2008). Anoikis: a necessary death program for anchorage-dependent cells. *Biochemical Pharmacology*.

[45] Gajewski T. F., Thompson C. B. (1996). Apoptosis meets signal transduction: elimination of a BAD influence. *Cell*.

[46] Scanga S. E., Ruel L., Binari R. C. (2000). The conserved PI3'K/PTEN/AKt signaling pathway regulates both cell size and survival in *Drosophila*. *Oncogene*.

[47] Wang K., Yuen S. T., Xu J. (2014). Whole-genome sequencing and comprehensive molecular profiling identify new driver mutations in gastric cancer. *Nature Genetics*.

[48] Reginato M. J., Mills K. R., Paulus J. K. (2003). Integrins and EGFR coordinately regulate the pro-apoptotic protein Bim to prevent anoikis. *Nature Cell Biology*.

[49] Cover T. L., Krishna U. S., Israel D. A., Peek R. M. (2003). Induction of gastric epithelial cell apoptosis by *Helicobacter pylori* vacuolating cytotoxin. *Cancer Research*.

[50] Galmiche A., Rassow J., Doye A. (2000). The N-terminal 34 kDa fragment of *Helicobacter pylori* vacuolating cytotoxin targets mitochondria and induces cytochrome c release. *The EMBO Journal*.

[51] Yamasaki E., Wada A., Kumatori A. (2006). *Helicobacter pylori* vacuolating cytotoxin induces activation of the proapoptotic proteins Bax and Bak, leading to cytochrome c release and cell death, independent of vacuolation. *The Journal of Biological Chemistry*.

[52] Valenzuela M., Bravo D., Canales J. (2013). *Helicobacter pylori*-induced loss of survivin and gastric cell viability is attributable to secreted bacterial gamma-glutamyl transpeptidase activity. *Journal of Infectious Diseases*.

[53] Nagasako T., Sugiyama T., Mizushima T., Miura Y., Kato M., Asaka M. (2003). Up-regulated Smad5 mediates apoptosis of gastric epithelial cells induced by *Helicobacter pylori* infection. *The Journal of Biological Chemistry*.

[54] Wandler A. M., Guillemin K. (2012). Transgenic expression of the *Helicobacter pylori* virulence factor CagA promotes apoptosis or tumorigenesis through JNK activation in Drosophila. *PLoS Pathogens*.

[55] Mimuro H., Suzuki T., Nagai S. (2007). *Helicobacter pylori* dampens gut epithelial self-renewal by inhibiting apoptosis, a bacterial strategy to enhance colonization of the stomach. *Cell Host and Microbe*.

[56] Oldani A., Cormont M., Hofman V. (2009). *Helicobacter pylori* counteracts the apoptotic action of its VacA toxin by injecting the CagA protein into gastric epithelial cells. *PLoS Pathogens*.

[57] Higashi H., Nakaya A., Tsutstumi R. (2004). *Helicobacter pylori* CagA induces Ras-independent morphogenetic response through SHP-2 recruitment and activation. *The Journal of Biological Chemistry*.

[58] Li N., Tang B., Zhu E.-D. (2012). Increased miR-222 in *H. pylori*-associated gastric cancer correlated with tumor progression by promoting cancer cell proliferation and targeting RECK. *FEBS Letters*.

[59] Bertaux-Skeirik N., Feng R., Schumacher M. A. (2015). CD44 plays a functional role in *Helicobacter pylori*-induced epithelial cell proliferation. *PLOS Pathogens*.

[60] Schlaermann P., Toelle B., Berger H. (2014). A novel human gastric primary cell culture system for modelling *Helicobacter pylori* infection in vitro. *Gut*.

[61] Wroblewski L. E., Piazuelo M. B., Chaturvedi R. (2015). *Helicobacter pylori* targets cancer-associated apical-junctional constituents in gastroids and gastric epithelial cells. *Gut*.

